# Evaluación del valor clínico del reflejo nauseoso en la disfagia orofaríngea neurogénica

**DOI:** 10.7705/biomedica.7009

**Published:** 2024-05-30

**Authors:** Juan Camilo Suárez, Sofía Illatopa, José Luis Echeverri, Santiago Zapata, José Bareño, Jorge Luis Sánchez

**Affiliations:** 1 Línea de Investigación en Discapacidad y Rehabilitación, Grupo de Salud Pública, Escuela de Ciencias de la Salud, Universidad Pontificia Bolivariana, Medellín, Colombia Universidad Pontificia Bolivariana Grupo de Salud Pública Escuela de Ciencias de la Salud Universidad Pontificia Bolivariana Medellín Colombia; 2 Facultad de Psicología y Medicina, Universidad CES, Medellín, Colombia Universidad CES Facultad de Psicología y Medicina Universidad CES Medellín Colombia; 3 Grupo de Investigación de Tecnología en Salud, Universidad CES, Medellín, Colombia Universidad CES Grupo de Investigación de Tecnología en Salud Universidad CES Medellín Colombia; 4 Departamento de Neurología, Facultad de Medicina, Universidad de Antioquia, Medellín, Colombia Universidad de Antioquia Departamento de Neurología Facultad de Medicina Universidad de Antioquia Medellín Colombia

**Keywords:** trastornos de la deglución, atragantamiento, examen neurológico, examen físico, deglutition disorders, gagging, neurologic examination, physical examination

## Abstract

**Introducción.:**

El reflejo nauseoso es un mecanismo de protección que impide que alimentos y agentes no deseados penetren en la vía aérea inferior. Usualmente, hace parte del examen físico de la deglución para detectar la disfagia orofaríngea, pero es un signo potencialmente ambiguo.

**Objetivo.:**

Evaluar el valor diagnóstico del reflejo nauseoso en pacientes con disfagia orofaríngea neurogénica y en pacientes sin ella.

**Materiales y métodos.:**

Se trata de un estudio observacional, analítico, en pacientes con disfagia orofaríngea neurogénica (casos) y en personas sin disfagia (controles), en el cual se evaluó por visualización directa la ausencia o la presencia del reflejo nauseoso de forma bilateral. Este resultado se ajustó por sexo, edad y otras variables de interacción.

**Resultados.:**

Se evaluaron 86 pacientes con disfagia orofaríngea neurogénica y 80 personas sin ella. En el examen físico de la deglución, la presencia del reflejo mostró una relación positiva con los pacientes (lado derecho: OR = 3,97; IC_95%_: 2,01-7,84; lado izquierdo: OR = 4,84; IC_95%_: 2,41-9,72), pero una asociación negativa con los controles. En ambos grupos, ni el sexo ni la edad, ni otras variables de interacción modificaron el reflejo nauseoso.

**Conclusiones.:**

La ausencia o la presencia del reflejo nauseoso no confirma ni excluye la existencia de una disfagia orofaríngea por causas neurológicas o neuromusculares; por lo tanto, no es recomendable que los profesionales de la salud se fíen del resultado de este reflejo. Los médicos tratantes deben ir más allá de una simple revisión del reflejo nauseoso, incluso en pacientes neurológicos en quienes se supone que debería estar ausente.

## Abstract (en)

**Introduction.:**

The gag reflex is a protection mechanism that prevents food and unwanted agents from entering the lower airways. It is usually part of the physical examination of swallowing to detect oropharyngeal dysphagia, but it is a potentially ambiguous sign.

**Objective.:**

To evaluate the diagnostic value of the gag reflex in patients with neurogenic oropharyngeal dysphagia and adults without it.

**Materials and methods.:**

We conducted an analytical observational study in patients with neurogenic oropharyngeal dysphagia (cases) and patients without dysphagia (controls). We evaluated the absence or presence of the reflex bilaterally, by direct visualization, and adjusted it according to sex, age, and other interaction variables.

**Results.:**

We included 86 patients with neurogenic oropharyngeal dysphagia and 80 control subjects. The gag reflex on swallowing physical examination showed a positive relationship with the patients (right side: OR = 3.97; 95 % CI: 2.01-7.84; left side: OR = 4.84; 95 % CI: 2.41-9.72), but a negative association with the control group. In both groups, neither sex, nor age, nor other interaction variables modified the gag reflex.

**Conclusions.:**

The gag reflex absence or presence does not confirm or exclude the existence of oropharyngeal dysphagia due to neurological and neuromuscular causes. Therefore, health professionals must not rely on this reflex. Clinicians must go beyond a simple reflex revision, even in neurological patients where it is supposed to be absent.

El reflejo nauseoso, también llamado reflejo del vómito o reflejo faríngeo, ocurre por la contracción de la base lingual, los músculos de la pared faríngea de forma bilateral y la elevación del paladar blando ^1^. Desde una óptica evolutiva, su función se resume en un mecanismo de protección, para evitar la asfixia o tragar objetos extraños ^1^ o alimentos potencialmente nocivos. Se presenta como una reacción somática en la que el cuerpo busca rechazar la ingestión de agentes indeseados ^2^ e impedir que penetren en la laringe y, posteriormente, en las vías aéreas inferiores.

El reflejo nauseoso se utiliza como prueba semiológica para observar la integridad o la alteración del tronco encefálico y declarar si hay muerte cerebral ^3^^,^^4^, en la valoración sensitiva y motora de los nervios glosofaríngeo y vago, y en la detección de disfagia durante el examen físico de la deglución ^1^.

En términos generales, la disfagia es un trastorno de la deglución ^5^ o la alteración del proceso de tragar, en el que hay problemas para mover de manera segura el bolo alimenticio desde la cavidad oral hasta el estómago ^6^. Clínicamente, se clasifica en orofaríngea y esofágica, pero también hay otras categorías según su etiología estructural, motora o funcional ^7^^,^^8^. En la disfagia orofaríngea, hay dificultad para mover el bolo alimenticio por compromiso de la fase oral, preparatoria oral o faríngea de la deglución; suele ocurrir por defectos estructurales -aquellas condiciones que originan o mantienen una luz estrecha en la cavidad oral, faríngea o esofágica- y funcionales, en los que hay deterioro de la fisiología de la deglución ^9^^,^^10^. La disfagia orofaríngea funcional puede tener causas neurológicas o neuromusculares, de ahí el término de disfagia orofaríngea neurogénica ^10^^,^^11^. Para este trastorno se reporta una incidencia mundial de 400.000 a 800.000 personas al año ^12^.

El reflejo nauseoso, en su parte aferente, está mediado por el nervio glosofaríngeo (IX nervio craneal) al estimular de forma táctil la faringe y el nervio trigémino (V nervio craneal) al tocar el paladar blando; en su parte eferente interviene el nervio vago (X nervio craneal).

Las raíces nerviosas de los nervios craneales IX y X se originan en el bulbo raquídeo y salen del cráneo a través del foramen yugular para llegar a ambos lados de la faringe e inervar su pared posterior, el tercio posterior de la lengua y el paladar blando ^13^.

Lo básico para desencadenar el reflejo es la estimulación con un objeto estéril y romo (depresor) de la pared faríngea posterior, de los pilares del istmo de las fauces y de la base lingual, sensación que es transmitida por el nervio glosofaríngeo -la vía aferente ipsilateral al núcleo solitario o gustativo-que se integra con el núcleo ambiguo. Este último es un núcleo de neuronas motoras atravesado por fibras nerviosas eferentes del nervio vago, que terminan en la musculatura faríngea y producen una contracción bilateral de los músculos faríngeos posteriores (músculos constrictores) para completar la aparición del reflejo ^1^. Durante el desarrollo del niño, el núcleo del tracto solitario se va adaptando fisiológicamente a porciones y texturas de alimentos cada vez más grandes, lo que hace que el reflejo nauseoso disminuya y no se desencadene por cualquier alimento sólido con el paso de los años ^1^.

Semiológicamente, se describe que la contracción faríngea ipsilateral al estímulo produce el reflejo nauseoso directo y la reacción contralateral al mismo produce el reflejo nauseoso consensuado ^1^. En la práctica, se ha reportado el hallazgo de hipersensibilidad al reflejo nauseoso en pacientes con trastorno de ansiedad, reflujo gastroesofágico o por tratamientos orales que estimulan continuamente la cavidad ^1^.

Al menos una de cada tres personas puede tener ausencia del reflejo nauseoso por habituación o influencia emocional, motivo por el cual los médicos tratantes no deberían confiar en la ausencia del reflejo como un predictor de aspiración en pacientes que han sufrido un accidente cerebrovascular ^1^. De esta manera, la ausencia o la presencia del reflejo nauseoso es un hallazgo potencialmente contradictorio y ambiguo. Sin embargo, entre el personal de salud existe la creencia de que la presencia del reflejo nauseoso en los pacientes es un predictor preciso del comportamiento de la deglución. Por esta razón, el reflejo se evalúa rutinariamente durante el examen físico de pacientes con trastornos de la deglución, a pesar de que la relación entre la capacidad para deglutir y el reflejo nauseoso no es totalmente clara ^14^^-^^16^.

El objetivo de este estudio fue evaluar el valor clínico del reflejo nauseoso en pacientes con disfagia orofaríngea neurogénica y en pacientes sin ella, mediante un examen físico neurológico centrado en la deglución.

## Materiales y métodos

Entre 2019 y 2021, se llevó a cabo un estudio observacional, analítico, de casos y controles, en pacientes adultos con disfagia orofaríngea neurogénica (casos) y adultos sin disfagia (controles), en el cual se evaluó el reflejo nauseoso como parte del examen físico centrado en la deglución.

Los criterios de selección de los casos fueron: edad mayor o igual a 18 años, sexo femenino o masculino, presencia de disfagia orofaríngea neurogénica de, al menos, un mes o más de evolución y diagnóstico de alguna enfermedad neurológica o neuromuscular que haya originado disfagia orofaríngea.

En el examen físico, se identificaron síntomas de disfagia mediante el instrumento *Eating Assessment Tool* (EAT-10) y se incluyeron aquellos con un puntaje total mayor o igual a tres puntos.

Se excluyeron los pacientes: con disfagia esofágica, mecánica, de propulsión o iatrogénica; con la piel de la región facial o cervical irradiada debido a algún tratamiento activo contra el cáncer; con procedimientos quirúrgicos en los últimos tres meses en la piel del cuello, o con demencia en fase avanzada que impida la comprensión de órdenes sencillas para masticar y tragar. También, se excluyeron aquellos pacientes en procedimientos de endodoncia en el momento, con malformaciones estructurales congénitas en la cavidad oral, la lengua o el cuello, con antecedentes de enfermedad de Sjögren o con enfermedad pulmonar obstructiva crónica con hipoxemia grave en el momento del examen.

Los criterios de selección del grupo control fueron: edad mayor o igual a 18 años, sexo femenino o masculino, sin diagnóstico de disfagia ni de enfermedades neurológicas centrales, periféricas o neuromusculares. Ausencia de comorbilidades como cáncer de cabeza y cuello, sin procedimientos quirúrgicos en las dos terceras partes inferiores del rostro o el cuello, ni uso de toxina botulínica. Se excluyeron aquellas personas sin disfagia que estuvieran en procedimientos de endodoncia en el momento o con presencia de malformaciones congénitas en cavidad oral, lengua o cuello, con antecedentes de enfermedad de Sjögren, compromiso cognitivo o enfermedad pulmonar obstructiva crónica.

Los pacientes se captaron en doce consultorios particulares de profesionales en fonoaudiología y deglución, en diez instituciones prestadoras de servicios de salud, en cinco centros de asistencia para adultos mayores y en tres fundaciones de pacientes ubicadas en el valle de Aburrá y San Nicolás (Antioquia, Colombia). Los sujetos sin disfagia se captaron en dos centros de estimulación, socialización y ocio para adultos y adultos mayores, en dos instituciones universitarias y en una junta de acción comunal, en el valle de Aburrá (Antioquia, Colombia). También, se captaron como controles, familiares de los pacientes.

Los criterios de selección para los casos fueron validados por una médica neuróloga, con experiencia asistencial en disfagia orofaríngea neurogénica, y el apoyo de una fonoaudióloga con entrenamiento en disfagia. Un médico especialista en rehabilitación neurológica con entrenamiento en disfagia seleccionó el grupo control sin disfagia.

Los dos grupos se tomaron de un estudio marco de cohorte que busca desarrollar una metodología clínica para el diagnóstico y el seguimiento de la disfagia orofaríngea mediante la integración de señales no invasivas y variables clínicas. Dicho estudio se llevó a cabo entre marzo del 2019 y diciembre del 2022, y contó con una muestra calculada de 76 pacientes con disfagia orofaríngea neurogénica y 76 personas de control, considerando una sensibilidad del Examen Clínico de la Deglución a la Cabecera del Paciente (ECD-CP) del 80 %, según lo reportado en la literatura ^17^.

La hipótesis era que la nueva metodología clínica tendría un incremento del 15 % en la sensibilidad comparada con la sola aplicación del ECD-CP (95 % en total), una potencia del 80 % y una confianza del 95 %. Para el desarrollo del presente trabajo, los pacientes y los individuos de control se sometieron al mismo examen físico de deglución y a la evaluación del reflejo nauseoso en el momento inicial del estudio de cohorte.

En los dos grupos se aplicó un ECD-CP que incluyó la evaluación del reflejo nauseoso de forma bilateral mediante un aplicador para estimular la orofaringe, la base lingual y el paladar blando. La persona debía estar en posición sedente, tranquila, sin toma de alimentos sólidos ni líquidos y con la cavidad oral iluminada con un fotóforo por parte del evaluador. Bajo visualización directa, el evaluador calificó el reflejo como ausente (sin elevación del paladar blando, sin contracción faríngea ni elevación del paladar blando, sin presencia de arcadas ni náuseas ni tos acompañante) o como presente (con elevación del paladar blando, contracción faríngea y elevación del paladar blando, con arcadas, náuseas o tos o sin ellas). Antes de estimular el reflejo nauseoso y después de hacerlo, y mediante la iluminación directa de la cavidad oral, el paladar blando y la pared posterior de la orofaringe, se observó si la úvula estaba centrada en la línea media o estaba desviada.

Se compararon los hallazgos del reflejo nauseoso (lado derecho e izquierdo) entre los casos y los controles, y luego se ajustaron con las variables de confusión "sexo" y "edad" y la variable de posible interacción "presencia de medicamentos que pueden modificar aspectos de la deglución" en ambos grupos. En el grupo de pacientes, las comparaciones se ajustaron con otras variables de potencial interacción como "tiempo de evolución de la etiología principal" (enfermedad neurológica o neuromuscular), "tiempo de evolución de la disfagia" y "autopercepción de síntomas de disfagia" mediante el instrumento EAT-10 (18). Se utilizó el EAT-10 validado para Colombia ^19^, impreso y diligenciado individualmente por el paciente o con apoyo de un cuidador o familiar cercano.

Las variables se clasificaron en cualitativas y cuantitativas, y se expresaron estadísticamente como magnitud y porcentaje, media y desviación estándar, o mediana y rango intercuartílico, según los supuestos de normalidad. Para las variables cualitativas se usaron razones de probabilidad (OR) con una significancia del 5 % y un intervalo de confianza del 95 %. Posteriormente, los análisis por subgrupos de interés se hicieron con las razones de probabilidad para tener la medición de la fuerza de asociación según el interés particular de la variable. El análisis se hizo con el *software* libre Jamovi, versión 2.5.5.

Por criterio estadístico, la edad se categorizó en menor de 60 años y en mayor o igual a 60 años, según la mediana (pacientes: 60,5 años, y controles: 61,5 años). Esta decisión también se apoyó en el ciclo de vida, donde la etapa de la vejez comienza a los 60 años ^20^^,^^21^. De acuerdo con el criterio de normalidad, el tiempo de evolución de la enfermedad neurológica o neuromuscular se categorizó en menor o igual a 4,5 años y mayor de 4,5 años; el tiempo de evolución de la disfagia, en menor o igual a 1,3 años y mayor de 1,3 años; y la autopercepción de los síntomas, mediante el puntaje del EAT-10 en menor o igual a 16 y mayor de 16 puntos. Las tres variables anteriores, tomadas solo en el grupo de pacientes, presentaron una distribución "no normal", por lo que se tomó como punto de corte a la mediana.

Los grupos farmacológicos, que según la literatura pueden modificar características de la deglución ^22^^-^^25^, se agruparon en la variable "presencia o ausencia de medicamentos en los casos y los controles". Los grupos farmacológicos identificados por anamnesis en los dos grupos fueron: barbitúricos, neurolépticos, antiinflamatorios no esteroideos, benzodiacepinas, relajantes musculares, antidepresivos tricíclicos y ansiolíticos.

Este estudio fue aprobado por el Comité de Ética de Investigación en Salud de la Universidad Pontificia Bolivariana (acta N° 7 del 1° de junio de 2017), el Comité de Ética de Investigación de la Fundación Hospitalaria San Vicente de Paúl (acta N° 35-2018, del 21 de diciembre de 2018) y el Comité de Ética en Investigación de la Clínica Somer (acta N°01-2019 del 8 de febrero de 2019). Se cuenta con el consentimiento informado de todas las personas que participaron en el estudio y se respetó su derecho a la privacidad.

## Resultados

**Figura 1:**
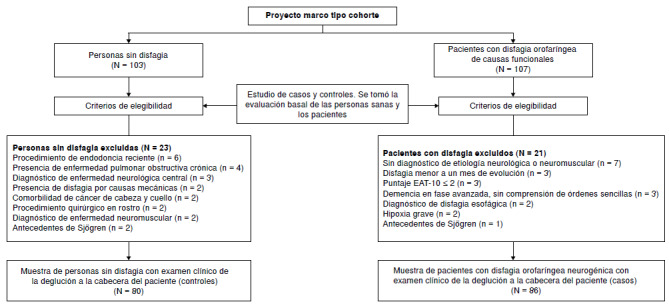
Flujograma de controles y pacientes del estudio. Entre paréntesis, aparece en detalle el número de personas control y de pacientes excluidos.

Entre los meses de marzo de 2019 y diciembre de 2021, se reclutaron y evaluaron 210 personas, 107 (50,95 %) con disfagia orofaríngea de causas funcionales y 103 (49,05 %) pacientes control. Al aplicar los criterios de selección, se logró una muestra final de 166 personas: 86 pacientes con disfagia orofaríngea neurogénica y 80 controles, todos con evaluación clínica de la deglución y el reflejo nauseoso (figura 1).

El sexo masculino representó el 53 % (88/166) de toda la muestra, siendo más frecuente en el grupo de pacientes, con el 59,3 % (51/86). En el grupo de controles, la mayoría fue de sexo femenino, con el 53,8 % (43/80). La mediana de edad en toda la muestra fue de 61 años (RIC: 51-68) (Shapiro-Wilk: p ≤ 0,001), la del grupo de pacientes de 60,5 años y la del grupo de controles de 61,5 años.

La comorbilidad más frecuente, en pacientes y en controles, fueron las enfermedades cardiovasculares, que incluyen hipertensión arterial sistémica, enfermedad coronaria, cardiopatías y arritmias. Las comorbilidades respiratorias -como enfermedad pulmonar obstructiva crónica, apnea obstructiva del sueño, asma y rinitis- y la diabetes, fueron más frecuentes en el grupo de pacientes. En el grupo de controles, las comorbilidades más frecuentes fueron las gastrointestinales, que incluyen reflujo gastroesofágico, gastritis y colon irritable, y el antecedente de COVID-19. En la totalidad de los controles y en la mayoría de los pacientes, la forma de alimentación en el momento del estudio fue la vía oral. En el cuadro 1 se muestran en detalle las características sociodemográficas y clínicas de la población de estudio.

**Cuadro 1:** Características de la muestra según condición de estudio

Características		Población	
		(N = 166)	
		Pacientes	Controles
		(n = 86)	(n = 80)
		n (%)	n (%)
Edad (años)	Promedio (DE)	56,8 (16,7)	58,9 (13,3)
	Mediana (RIC)	60,5 (48,3-68)	58,9 (13,3)
	Rango	18-84	22-90
Sexo	Femenino	35 (40,7)	43 (53,8)
	Masculino	51 (59,3)	37 (46,3)
Comorbilidades	Cardiovascular	35 (40,7)	24 (30,0)
	Tiroidea	9 (10,5)	11 (13,7)
	Dislipidémica	9 (10,5)	11 (13,7)
	Gastrointestinal	8 (9,3)	10 (12,5)
	Reumatológica	9 (10,5)	8 (10,0)
	Diabética	11 (12,8)	5 (6,2)
	Respiratoria	16 (18,6)	3 (3,7)
	Emocional	6 (7)	3 (3,7)
	COVID-19	5 (5,8)	12 (15,0)
	Oral	81 (94,2)	80 (100)
	Gastrostomía	2 (2,3)	0 (0)
	Mixta	3 (3,5)	0 (0)
Medicación que pueda modificar la deglución, presente en el momento de la evaluación		26 (30,2)	6 (7,5)

DE: desviación estándar; RIC: rango intercuartílico

En ambos grupos se logró documentar, al inicio del estudio, los medicamentos que pudieran modificar la deglución, cuya presencia fue más frecuente en el grupo de pacientes. Entre estos, se identificaron cinco grupos farmacológicos: neurolépticos (18,6 %; 16/86), antiinflamatorios no esteroideos (7 %; 6/86), benzodiacepinas (3,5 %; 3/86), relajantes musculares y antidepresivos tricíclicos (cada uno: 1,2 %; 1/86). En los individuos control, se identificaron dos grupos farmacológicos: antiinflamatorios no esteroideos (6,3 %; 5/80) y antidepresivos tricíclicos (1,3 %; 1/80).

En el grupo de pacientes, la etiología neurogénica de la disfagia orofaríngea fue por causas neurológicas centrales en el 88,4 % (76/86) y neuromusculares en el 11,6 % (10/86) de los sujetos.

Las causas neurológicas centrales incluían: enfermedad de Parkinson (23,7 %; 18/76); esclerosis lateral amiotrófica (18,4 %; 14/76); accidente cerebrovascular (17,1 % 13/76); esclerosis múltiple (13,2 %; 10/76); demencias (9,2 %; 7/76); traumatismo craneoencefálico (6,6 %; 5/76); parálisis cerebral infantil (5,3 %; 4/76); ataxia (3,9 %; 3/76) y otras (2,6 %; 2/76). En la categoría de "otras" se incluyeron talamotomía por la observación de movimientos anormales en un paciente e hidrocefalia secundaria a neurocisticercosis en otro. Las causas neuromusculares identificadas fueron distrofia muscular (50 %; 5/10); dermatomiositis (20 %; 2/10); neuropatía (20 %; 2/10) y miastenia grave (10 %; 1/10). En el grupo de pacientes, la mediana de años de evolución de la causa neurogénica (central o neuromuscular) de la disfagia orofaríngea fue de 4,5 años (cuadro 2), mientras que la evolución general de la disfagia orofaríngea como tal fue de 1,33 años.

En cuanto a los síntomas de disfagia, los más frecuentes fueron la sensación de comida pegada luego de tragar, la sensación de comida atascada en el cuello, la tos después de tragar, la sensación de ahogo luego de tragar y la dificultad para masticar. La mediana de puntaje del instrumento EAT-10 fue de 16. En el cuadro 2 se detallan las características clínicas de la disfagia orofaríngea en el grupo de pacientes.

**Cuadro 2:** Características clínicas de la disfagia orofaríngea neurogénica en los pacientes del estudio

Características		Pacientes	
		(n = 86)	
Refiere problemas para iniciar o comenzar a tragar		28	(32,6)
Sensación de comida pegada segundos después de tragar		77	(89,5)
Sitio de sensación de comida atascada	Boca	14	(18,2)
	Cuello	55	(71,4)
	Tórax	1	(1,3)
	No especificado	7	(9,1)
Tos antes de tragar		15	(17,4)
Tos después de tragar		63	(73,3)
Sensación de ahogo después de tragar		40	(46,5)
Regurgitación nasal de comida		11	(12,8)
Dificultad para masticar		35	(40,7)
Tipo de alimento con mayor sensación de dificultad para tragar	Líquidos	16	(18,6)
	Sólidos	52	(60,5)
	Ambos	18	(20,9)
Puntaje total EAT-10	Promedio (DE)	17,3	(8,57)
	Mediana (RIC)	16	(10,5-21,0)
	Rango	3-40	
Años de evolución de la enfermedad causante de disfagia	Promedio (DE)	9,75 (12,2)	
	Mediana (RIC)	4,5	(1,0-16,5)
	Rango	0,1-65,0	
Años de evolución de la disfagia en la persona	Promedio (DE)	3,18	(5,48)
	Mediana (RIC)	1,33	(0,62-3,0)
	Rango	0,08-31,0	

DE: desviación estándar; RIC: rango intercuartílico; EAT-10: *Eating Assessment Tool*

La presencia del reflejo nauseoso derecho e izquierdo durante el examen físico de la deglución, se observó en pacientes con disfagia orofaríngea neurogénica y sujetos control (sin disfagia). Sin embargo, se encontró una relación positiva del reflejo con el grupo de pacientes y una asociación negativa con el de controles, es decir, la presencia del reflejo nauseoso en el examen físico aumenta la probabilidad de tener disfagia orofaríngea neurogénica, mientras que la ausencia del reflejo nauseoso está más relacionada con no padecer disfagia (cuadro 3).

**Cuadro 3:** Comparación de hallazgos del reflejo nauseoso y la posición de la úvula, durante el examen físico, en pacientes y controles

Variables		Población		OR	p	IC_95%_
		(N = 166)				
		Pacientes	Controles			
		(n = 86)	(n = 80)			
		n (%)	n (%)			
Reflejo nauseoso derecho	Presente	68 (79,1)	39 (48,8)	3,97	< 0,001*	2,01-7,84
	Ausente	18 (20,9)	41 (51,2)			
Reflejo nauseoso izquierdo	Presente	70 (81,4)	38 (47,5)	4,84	< 0,001*	2,41- 9,72
	Ausente	16 (18,6)	42 (52,5)			
Úvula desviada	Sí	8 (9,3)	0 (0)	17,4	0,007**	0,99-307
	No	78 (90,7)	80 (100)			

OR: *odds ratio;* IC: intervalo de confianza

* X^2^

** test exacto de Fisher

En el grupo control, las variables de confusión "sexo" y "edad", y la variable de potencial interacción "presencia de medicamentos que modifican la deglución", no mostraron diferencias estadísticamente significativas, ni asociación con el hallazgo de reflejo nauseoso ausente (cuadro 4).

**Cuadro 4:** Comparación del reflejo nauseoso por lado, en el grupo control, por sexo, edad y presencia de medicamentos

Variable		Reflejo nauseoso lado derecho		OR	p	IC_95%_
		Ausente	Presente			
		(n = 41)	(n = 39)			
		n (%)	n (%)			
Sexo	Femenino	23 (56,1)	20 (51,3)	1,21	0,666*	0,50-2,93
	Masculino	18 (43,9)	19 (48,7)			
Edad (años)	< 60	18 (43,9)	14 (35,9)	1,40	0,465*	0,57-3,43
	≥ 60	23 (56,1)	25 (64,1)			
Presencia de medicación que modifique la deglución	Sí	2 (4,9)	4 (10,3)	0,45	0,426**	0,07-2,60
	No	39 (95,1)	35 (89,7)			
Variable		Reflejo nauseoso lado izquierdo		OR	p	IC_95%_
		Ausente	Presente			
		(n = 42)	(n = 38)			
Sexo	Femenino	22 (52,4)	21 (55,3)	0,89	0,796*	0,37-2,15
	Masculino	20 (47,6)	17 (44,7)			
Edad (años)	< 60	19 (45,2)	13 (34,2)	1,59	0,315*	0,64-3,93
	≥ 60	23 (54,8)	25 (65,8)			
Presencia de medicación que modifique la deglución	Sí	1 (2,4)	5 (13,2)	0,16	0,097**	0,01-1,45
	No	41 (97,6)	33 (86,8)			

OR: *odds ratio;* IC: intervalo de confianza

* X^2^

** test exacto de Fisher

En el grupo de pacientes, las variables de confusión "sexo y edad", y las variables de potencial interacción como "presencia de medicamentos que modifican la deglución", "tiempo de evolución del diagnóstico de la causa neurogénica de tipo central o neuromuscular", "tiempo de la disfagia" y "puntaje del instrumento EAT-10", no mostraron diferencias estadísticamente significativas, ni asociación con la ausencia de reflejo nauseoso (cuadro 5).

**Cuadro 5:** Comparación del reflejo nauseoso por lado, en el grupo de pacientes, por sexo, edad, presencia de medicamentos, tiempo de evolución del diagnóstico de disfagia y puntaje EAT-10

Variable		Reflejo nauseoso lado derecho		OR	p	IC_95%_
		Ausente	Presente			
		(n = 18)	(n = 68)			
		n (%)	n (%)			
Sexo	Femenino	8 (44,4)	27 (39,7)	1,21	0,716*	0,42-3,47
	Masculino	10 (55,6)	41 (60,3)			
Edad (años)	< 60	6 (33,)	34 (50)	0,50	0,207*	0,16-1,49
	≥ 60	12 (66,7)	34 (50)			
Autopercepción de síntomas por EAT-10	≤ 16 puntos	9 (50)	37 (54,4)	0,83	0,739*	0,30-2,37
	> 16 puntos	9 (50)	31 (45,6)			
Presencia de medicación que modifique la deglución	Sí	6 (33,3)	20 (29,4)	1,20	0,747*	0,39-3,64
	No	12 (66,7)	48 (70,6)			
Tiempo de evolución de la causa neurogénica§	≤ 4,5 años (n= 40)	9 (60)	31 (47,7)	1,65	0,390*	0,52-5,15
	> 4,5 años (n= 40)	6 (40)	34 (52,3)			
Tiempo de evolución de la disfagia^δ^	≤ 1,3 años (n= 42)	6 (35,3)	36 (54,5)	0,45	0,157*	0,15-1,37
	> 1,3 años (n = 41)	11 (64,7)	30 (45,5)			
Variable		Reflejo nauseoso lado izquierdo		OR	p	IC_95%_
		Ausente	Presente			
		(n=16)	(n=70)			
		n (%)	n (%)			
Sexo	Femenino	7 (43,8)	28 (40)	1,17	0,783*	0,39-3,50
	Masculino	9 (56,3)	42 (60)			
Edad (años)	< 60	6 (37,5)	34 (48,6)	0,63	0,423*	0,21-1,94
	≥ 60	10 (62,5)	36 (51,4)			
Autopercepción de síntomas por EAT-10	≤ 16 puntos	7 (43,8)	39 (55,7)	0,62	0,387*	0,21-1,85
	> 16 puntos	9 (56,3)	31 (44,3)			
Presencia de medicación que modifica deglución	Sí	5 (31,3)	21 (30)	1,06	1**	0,39-3,43
	No	11 (68,8)	49 (70)			
Tiempo de evolución de la causa neurogénica^§^	≤ 4,5 años (n	7 (53,8)	33 (49,3)	1,20	0,762*	0,36-3,95
	> 4,5 años (n	6 (46,2)	34 (50,7)			
Tiempo de evolución de la disfagia^δ^	≤ 1,3 años (n	7 (46,7)	35 (51,5)	0,82	0,736*	0,23-2,53
	> 1,3 años (n	8 (53,3)	33 (48,5)			

OR: *odds ratio.* IC: intervalo de confianza del 95 %

* X^2^

** test exacto de Fisher

§ El tiempo de evolución de la enfermedad neurogénica no se documentó en seis pacientes.

^δ^ El tiempo de evolución de la disfagia no se documentó en tres pacientes.

## Discusión

El presente estudio se centró en comparar los hallazgos del reflejo nauseoso entre una muestra representativa de pacientes adultos con disfagia orofaríngea neurogénica (casos) y de individuos sin disfagia (controles). Los resultados son contradictorios porque el reflejo se presentó en pacientes y también en individuos control. Sin embargo, se encontró una asociación positiva, estadísticamente significativa, entre el reflejo nauseoso y los pacientes (OR = 3,97 para el reflejo del lado derecho y OR = 4,84, del lado izquierdo, con intervalos de confianza estrechos), pero negativa, entre la ausencia de dicho reflejo y los sujetos control.

Estos resultados cuestionan el valor clínico de este reflejo en la evaluación de pacientes con disfagia orofaríngea neurogénica y se suman a los de varios estudios con hallazgos similares: el reflejo nauseoso en pacientes con disfagia tiene resultados ambiguos.

El reflejo nauseoso puede estar presente o ausente, tanto en personas sin disfagia como en pacientes con situaciones neurológicas y neuromusculares que hayan desencadenado disfagia orofaríngea. Si bien la alteración del reflejo nauseoso se relaciona con la etiopatogenia de base (e.g., por afección del nervio glosofaríngeo o enfermedades que alteren el bulbo raquídeo), los resultados del presente trabajo muestran que la ambigüedad de su presencia también puede ocurrir cuando se agrupan varias causas, como la disfagia orofaríngea neurogénica. En este estudio, la ausencia del reflejo nauseoso (independiente del lado) se identificó durante el examen físico en ambos grupos, pero con mayor frecuencia en el de control, mientras que su presencia fue más frecuente en el grupo de pacientes.

El resto de los resultados obtenidos sugieren que ni el sexo, ni la edad ni los medicamentos que puedan modificar aspectos de la deglución, afectan la presencia o ausencia del reflejo nauseoso en individuos control o con disfagia orofaríngea neurogénica. De igual manera, características propias de los pacientes con disfagia orofaríngea neurogénica, como el tiempo de evolución de la enfermedad o la etiología principal, el tiempo de evolución y la autopercepción de síntomas de disfagia (mediante el instrumento EAT-10), no parecen modificar la presencia o ausencia del reflejo nauseoso, independientemente del lado (derecho o izquierdo).

El reflejo nauseoso puede estar ausente en adultos sin disfagia, por lo que su uso como herramienta diagnóstica es controversial, por ejemplo, en casos de pacientes que han sufrido algún accidente cerebrovascular. En un estudio en el que se evaluaron 140 participantes sin disfagia (entre jóvenes y adultos mayores) para comprobar la presencia del reflejo nauseoso y la sensación faríngea, se encontró que el 37 % de los sujetos examinados no presentaba el reflejo. Este resultado apoya la poca utilidad de este signo en la evaluación del riesgo de aspiración en pacientes con accidentes cerebrovasculares, por su escaso valor predictivo ^26^.

Leder cuestionó el rol del reflejo nauseoso en la protección contra la aspiración en pacientes con disfagia en un estudio con 69 casos. El investigador encontró que nueve pacientes no tenían reflejo nauseoso y, de estos, siete tenían movimiento normal del velo del paladar, por lo que no se pudo demostrar que el reflejo nauseoso fuera un predictor clínico de disfagia ^14^. En los pacientes aquí evaluados, la desviación de la úvula no se asoció con el grupo de pacientes, ni con el de controles.

En la evaluación de un grupo de 10 pacientes con traumatismo craneoencefálico grave y sospecha de aspiración mediante videofluoroscopia, solo se encontró alteración en los reflejos palatal y nauseoso en el 30 % de los pacientes ^27^. Se menciona que la ausencia del reflejo nauseoso o alteraciones en la sensibilidad laringofaríngea se asocian con dificultades deglutorias como la disfagia, pero estos dos signos evaluados individualmente no parecen predecir el riesgo de aspiración ^28^^,^^29^.

En Latinoamérica, en un estudio chileno retrospectivo que incluyó 629 registros de pacientes hospitalizados con disfagia, en su mayoría con etiología neurogénica (68,45 %), solo reportaron dos casos de alteración del reflejo nauseoso ^30^.

Existen otros trabajos que apoyan la ausencia del reflejo nauseoso como herramienta clínica en la evaluación de la disfagia: Ramsey *et al.* examinaron 242 pacientes con antecedente de accidentes cerebrovasculares y determinaron la ausencia del reflejo en el 38,6 % de los pacientes con disfagia y el 3,5 % de aquellos sin disfagia. Estos autores sostienen que el reflejo nauseoso es específico para la detección de disfagia en pacientes con accidentes cerebrovasculares, pero menos sensible que la "evaluación de la deglución a la cabecera" ^31^.

Clínicamente, la ausencia del reflejo nauseoso se ha utilizado para evaluar la disfagia orofaríngea por diferentes causas. Sin embargo, se ha obervado que dicha ausencia no es infrecuente; de hecho, se ha documentado en pacientes con antecedentes de tabaquismo ^1^ y edad avanzada, sin enfermedades neurológicas o funcionales, probablemente relacionado con el deterioro de la integridad de las vías aferentes del reflejo ^26^.

Considerando los antecedentes médicos y las características semiológicas de los individuos sin disfagia y los pacientes con disfagia incluidos en este trabajo, los resultados de otros estudios y los aspectos fisiológicos propios del reflejo nauseoso, no es posible postular que un solo factor sea el principal responsable de la ausencia del reflejo en personas sanas y de su mayor frecuencia en pacientes con disfagia orofaríngea neurogénica. La ausencia o presencia del reflejo nauseoso, posiblemente, sea el resultado de interacciones dinámicas entre la mucosa orofaríngea, las aferencias de los nervios glosofaríngeo y trigémino, las eferencias transmitidas por el nervio vago, las modulaciones en varios núcleos del tallo cerebral y los mecanismos de adaptación (inhibición o facilitación) mediados por estructuras corticales y subcorticales. También, puede deberse a posibles adaptaciones del reflejo en individuos sanos a lo largo de su experiencia de comer y en pacientes con disfagia orofaríngea neurogénica, como mecanismo de protección en medio de una deglución alterada.

La ausencia o la presencia del reflejo nauseoso no confirma ni excluye la existencia de una disfagia orofaríngea de origen neurológico o neuromuscular. De manera que, a partir de los resultados obtenidos, concordantes con los de otros estudios, no es recomendable que los profesionales de la salud se fíen del resultado clínico del reflejo nauseoso, ni de la posición de la úvula, para diagnosticar disfagia orofaríngea, especialmente cuando se trate de pacientes con causas neurológicas o neuromusculares. A los médicos tratantes se les sugiere ir más allá de una simple revisión del reflejo nauseoso, incluso, en pacientes neurológicos en quienes se supone que debería estar ausente.

La disfagia orofaríngea neurogénica es un síndrome multietiológico con varios patrones fenotípicos según la enfermedad neurológica ^32^ o neuromuscular de base. La valoración formal de la deglución y la disfagia mediante una evaluación clínica de la deglución a la cabecera del paciente (ECD-CP) es el primer paso diagnóstico ^33^; sin embargo, la sospecha de disfagia durante el examen físico debe confirmarse mediante pruebas instrumentales (de referencia), como la videofluoroscopia ^15^.

La evaluación clínica de la deglución debe incluir, al menos, la revisión de los nervios craneales, un examen sensorial y motor de la cavidad oral, y una evaluación de la deglución orofaríngea usando diferentes alimentos y líquidos ^34^, yendo más allá de una simple revisión del reflejo nauseoso.
